# Liddle’s syndrome in an African male due to a novel frameshift mutation in the beta-subunit of the epithelial sodium channel gene

**DOI:** 10.5830/CVJA-2017-012

**Published:** 2017

**Authors:** Robert Freercks, Jason Ensor, Clarise Weimers-Willard, Surita Meldau, Robert Freercks, Erika Jones, Jason Ensor, Brian Rayner

**Affiliations:** Division of Nephrology and Hypertension, Livingstone Hospital, Port Elizabeth, South Africa; Division of Nephrology and Hypertension, Livingstone Hospital, Port Elizabeth, South Africa; Division of Nephrology and Hypertension, Livingstone Hospital, Port Elizabeth, South Africa; Division of Chemical Pathology, University of Cape Town and National Health Laboratory Service, Cape Town, South Africa; Department of Medicine, Division of Nephrology and Hypertension, University of Cape Town, Cape Town, South Africa; Department of Medicine, Division of Nephrology and Hypertension, University of Cape Town, Cape Town, South Africa; Department of Medicine, Division of Nephrology and Hypertension, University of Cape Town, Cape Town, South Africa; Department of Medicine, Division of Nephrology and Hypertension, University of Cape Town, Cape Town, South Africa

**Keywords:** hypertension, Liddle’s, Africa, amiloride, resistant hypertension, hypokalaemia, low renin

## Abstract

Resistant hypertension is a common clinical problem in South Africa and is frequently associated with low renin and aldosterone levels, especially in black Africans. In South Africa, novel variants in the epithelial sodium channel (ENaC) have been described to be associated with varying degrees of hypokalaemia and hypertension due to primary sodium retention. We report here a case of Liddle’s syndrome due to a novel c.1709del11 (p.Ser570Tyrfs*20) deletion in the beta-subunit of the ENaC in a young black African male. We discuss the likely pathogenesis of hypertension in this setting as well as the treatment options available in South Africa aimed at the ENaC. This case highlights the need for vigilance in detecting and appropriately treating low-renin and low-aldosterone hypertension in view of the frequency of the described variants of the ENaC channel in our cuntry. Specific therapy such as amiloride should be made more widely available.

## Case report

An 18-year-old Xhosa-speaking South African male was referred to the Livingstone Hospital renal unit for evaluation. He first presented at the age of 17 years to a nearby hospital emergency unit with a headache. He was not on any chronic or over-thecounter medications, did not consume liquorice, ethanol or traditional medications and was a non-smoker. He was noted to be hypertensive, with a blood pressure of 216/114 mmHg and hypokalaemic, with a serum potassium level of 2.9 mmol/l (see Table 1), but he left without treatment.

Eight months later he was seen at the same unit with a similar presentation and was admitted for further investigations and treatment. Despite multiple antihypertensive interventions, his blood pressure remained uncontrolled. At discharge he was commenced on the following medications: enalapril 10 mg 12 hourly, amlodipine 10 mg daily, furosemide 40 mg twice daily, atenolol 25 mg daily, hydrallazine 50 mg twice daily and hydrochlorothiazide 25 mg daily.

He was referred to our unit, and one month later his blood pressure was 179/118 mmHg in the left arm and 182/113 mmHg in the right arm, despite adherence to the treatment regime. He looked well, had a regular pulse rate of 59 beats/min and weighed 68 kg. The patient gave a history of experiencing frequent headaches associated with muscle fatigue, but no myalgias. The muscle fatigue was worse at times when the headache was present. He also described exertional dyspnoea but no spells suggestive of phaeochromocytoma. Both his parents were hypertensive and his father had died of an uncertain cause before the age of 50 years.

On cardiovascular examination, all pulses were present. There Division of Nephrology and Hypertension, Livingstone was no radiofemoral delay or any bruits. The cardiac apex beat was forceful and laterally displaced with an associated fourth heart sound. He had normal secondary sexual characteristics, and retinal examination revealed markedly reduced arteriolar diameter and arteriovenous nicking. The urine dipstick was normal as was an ultrasound of the kidneys, ureters and bladder.

Chest radiography showed a bulky heart shadow and electrocardiogram confirmed left ventricular hypertrophy with a Sokolow–Lyon score of 88 mV. Laboratory investigationsconfirmed the hypokalaemia and his serum potassium level wasnow 2.7 mmol/l, associated with a supressed renin level of 6.0 mIU/l and aldosterone of 48.6 pmol/l (Table 1). Unfortunately, arterial blood gas analysis was not performed prior to treatment.

**Table 1:** Blood results and blood pressure readings

*Test*	*02/2014*	*10/2015*	*11/2015*	*01/2016*	*03/2016*	*06/2016*	*08/2016*
Sodium, mmol/l	142		140		139	136	
Potassium, mmol/l	2.9	3.4	2.7	3.5	3.3	3.7	3.7
Creatinine, umol/l	88	220	150	144	134	153	128
Renin, mIU/l (9.2–69.7)	6.0				Amiloretic added 03/2016		
Aldosterone, pmol/l (94–757)	48.6						
Office BP, mmHg	216/114	220/120	179/118	173/101	181/121	162/91	142/100

Based on this presentation, genetic testing for the locally prevalent c.1815G>A (p.R563Q)^1^ Liddle’s syndrome-associated genetic variant in the SCNN1B gene was requested, and his treatment was intensified to include doxazosin as no amiloridecontaining medications were available at the time. His blood pressure remained uncontrolled and the c.1815G>A (p.R563Q) variant screen was negative.

**Fig. 1.**
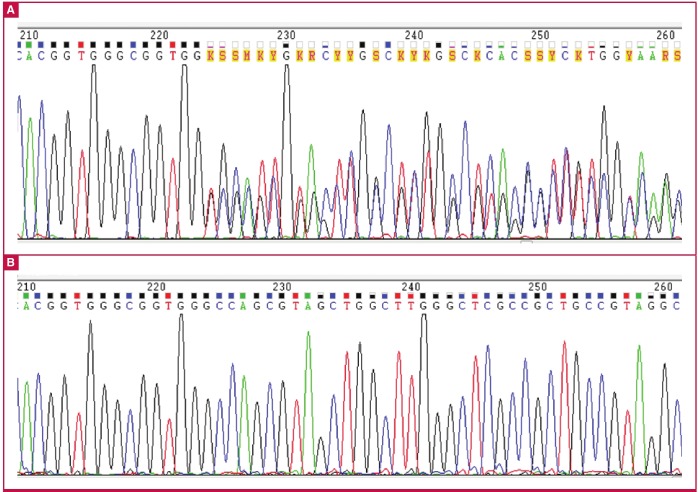
Sequence electropherogram showing the deletion in exon 13 of the SCNN1B gene (A), compared to a control sequence (B).

Due to his persistent hypokalaemia, hypertension and supressed renin and aldosterone levels, sequencing of exon 13 of the beta-chain of the epithelial sodium channel was pursued. A novel heterozygous 11bp deletion in the SCNN1B gene was detected (Fig. 1A, case and 1B, control). The mutation causes a frame shift in exon 13 of the gene, resulting in a premature stop codon and truncated protein product.

His family was called in for screening. We have been unable to see his mother who lives in a rural area distant from our clinic. He has two brothers and one sister who are from the same parents. His younger brother (11 years) and his older sister (21 years) were both well and had normal blood pressures. His older brother (25 years) was found to have a raised blood pressure of 141/77 mmHg on ambulatory monitoring but was overweight with signs of insulin resistance and he had a body mass index of 35.1 kg/m^2^. All three siblings tested negative for the mutation seen in this patient and had potassium, renin and aldosterone levels in the normal range.

As amiloride is not registered in South Africa and is only available in combination with hydrochlorothiazide (5 mg amiloride; 50 mg hydrochlorothiazide) (Amiloretic, Aspen Pharmacare, South Africa), he was started on the latter. Over the next few months his blood pressure improved significantly, with office readings of 162/91, 139/84, 190/113 and 142/100 mmHg. He still requires concomitant treatment with enalapril 10 mg daily, amlodipine 10 mg daily, atenolol 50 mg daily, doxazosin XL 8 mg daily and potassium replacement. On one occasion, after running out of tablets for three days, his blood pressure was 221/157 mmHg.

His creatinine level has remained elevated but he has had no further headaches. However, potassium levels remain low and we suspect that higher doses of amiloride are needed. This will be introduced, pending local regulatory approval.

## Discussion

Low-renin hypertension has been found to be more common in people of black African origin.^2^ Furthermore, in South Africa, low renin was found to be associated with low aldosterone levels in patients with resistant hypertension.

The epithelial sodium channel is the final and rate-limiting step in sodium reabsorption. Liddle’s syndrome was described in a family that presented with hypertension and hypokalaemia associated with metabolic alkalosis.^3^ The genetic variant was found to be a truncating mutation of the beta-subunit of the epithelial sodium channel. Truncation of the carboxy terminal of the betasubunit results in impaired internalisation of the channel with ongoing sodium and water reabsorption, resulting in hypertension.

Aberations due to activating mutations within the carboxyl terminal region of the epithelial sodium channel (ENaC) betaor alpha-subunits are known to cause Liddle’s syndrome. Such mutations negatively affect targeting of the protein products for endocytic degradation, which in turn leads to increased sodium reabsorption and resultant hypertension.^4^ The mechanism is thought to be due to interference with PPPY-targeting motifs needed for recognition by NEDD4 ubiquitin ligases

Multiple variants in the SCNN1B gene have been reported, many causing complete Liddle’s syndrome. Some mutations affect the PY motif, others truncate the carboxy terminal. In the case of single-nucleotide polymorphisms that do not affect the PY motif, the full Liddle’s phenotype is not usually present.^5^,^6^ A variant (R563Q, c.1815G>A) of the beta-subunit of the epithelial sodium channel was found frequently in patients with resistant hypertension and with low renin and aldosterone levels.^7^ This variant was described to result in the full Liddle’s phenotype during pregnancy;^8^ and in black South African hypertensive patients, the prevalence was 5.9% versus 1.7% in normotensives.^1^

Unlike the c.1815G>A (p.R563Q) variant, which affects a single amino acid in the protein product, the c.1709del11 (p.Ser570Tyrfs*20) deletion, seen in our patient (Fig. 1A), causes a frame shift, resulting in complete mistranslation from this point onwards and ultimately resulting in a premature stop codon 20 amino acids downstream. This mutation results in loss of the PPPY motif, impairing ENaC degradation, and resulting in unopposed sodium and water reabsorption.

As neither parent could be tested and no siblings were found to carry this variant, it is impossible to say whether this is a new or inherited variant. However, both parents were hypertensive and it is likely that one of them are/were affected. Ideally the mother of this patient and/or any living paternal siblings should be tested. The mild hypertension present in the older brother can be explained on the basis of him having the metabolic syndrome phenotype.

In patients with Liddle’s syndrome, treatment requires inhibition of the sodium channel. There are two agents available for this: triamterene and amiloride. In the intial stages of hypertension, inhibition of the sodium channel can completely reverse the clinical effects. After prolongeduncontrolled hypertension, the vascular consequences can result in hypertension that is more difficult to treat.

Neither amiloride nor triamterene are licenced in South Africa, which impacts negatively on treatment. Amiloride is only available in combination with large doses of hydrochlorothiazide (5 mg amiloride/50 mg hydrochlorothiazide), which is not ideal in the setting of hypokalaemia. Our patient has responded well to this treatment, although he is not yet fully controlled and most likely requires a higher dose of amiloride.

Amiloride 10 mg can be issued on an individualised basis under section 21 drug use but availability is limited and extremely expensive. Due to the frequency of described variants of the SCNN1B gene in South Africa, specific therapy targeting the epithelial sodium channel should be made more widely available.

## Conclusion

We have described a novel c.1709del11 (p.Ser570Tyrfs*20) deletion in the beta-subunit of the ENaC associated with Liddle’s syndrome in a young black African male. Due to this frameshift mutation, the defect is likely causative of the hypertension and therapy should be targeted at inhibition of ENaC. Unfortunately, this is not easily achievable in our context and treatment of such patients is therefore severely hampered in South Africa.
